# Detection of *Theileria parva* in tissues of cattle undergoing severe East Coast fever disease show significant parasite DNA accumulation in the spleen

**DOI:** 10.1016/j.vetpar.2016.11.012

**Published:** 2016-12-15

**Authors:** Cassandra L. Olds, Tasha Paul, Glen A. Scoles

**Affiliations:** a Washington State University, Department of Veterinary Microbiology and Pathology, P.O. Box 647040, Pullman, WA 99164-6630, United States; b Animal Disease Research Unit, USDA Agricultural Research Service, Pullman, WA 99164-6630, United States

**Keywords:** *Theileria parva*, Nested PCR, Quantitative PCR, Spleen

## Abstract

Infiltration and proliferation of *Theileria parva* infected lymphocytes in bovine host lymphoid organs is one of the hallmarks of *T. parva* infection. The relative abundance of parasites within infected host tissues, both lymphoid and non-lymphoid is however unknown. Using quantitative PCR, we have shown that significantly higher concentrations of *T. parva* DNA are detected in the spleens of cattle undergoing severe disease compared to other organs.

## Highlights

• This is the first quantification of *T. parva* parasite DNA in the organs of infected cattle.
• Significant accumulation of *T. parva* parasite DNA occurs in the spleen of cattle during severe disease.
• *T. parva* DNA is quantifiable in the brain of cattle during severe infection.

## Graphical abstract

**Figure:**
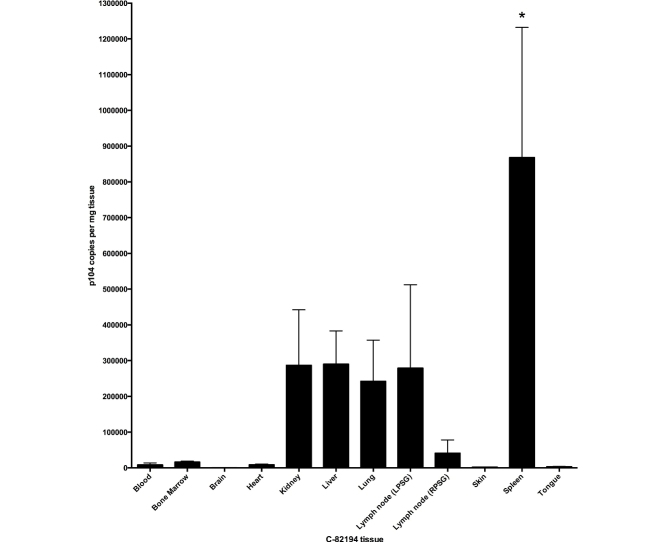

*Theileria parva*, the causative agent of East Coast fever (ECF) is an economically important tick-borne pathogen of cattle in Africa. Transmitted by the three-host tick, *Rhipicephalus appendiculatus,* the disease occurs throughout East, Central, and Southern Africa. Although disease susceptibility and severity varies between cattle species and individual animals, it is largely dependent on the number of sporozoites inoculated into the cattle host (reviewed by Norval et al., 1992, Bishop et al., 2004, Minjauw and McLeod, 2003). During tick feeding, sporozoite stage parasites are released from tick salivary glands into the feeding lesion in the host skin (Konnai et al., 2006, Konnai et al., 2007). Sporozoites then rapidly invade B and T cells (either CD4+, CD8+ or CD4x/CD8x) through a zippering process involving parasite and host cell molecules (Baldwin et al., 1988, Morrison et al., 1996, Shaw, 1996, Tindih et al., 2012). Once within a host lymphocyte, multi-nucleated shizont stage parasites form and induce clonal proliferation of the host cell (lymphoblasts) in which each daughter host cell receives at least one copy of parasite (Hulliger et al., 1964). These shizont infected lymphoblasts are rapidly disseminated from the site of infection to lymphoid organs throughout the body and parasites can be detected in the spleen and pre-scapular lymph nodes on the same day as sporozoite inoculation at the ear (Shatry et al., 1981). The presence of *T. parva* infected host cells in lymphoid organs during acute infection has been well described although infiltration into non-lymphoid tissues has received less attention (Kimeto, 1981, Mbassa et al., 2006). In an effort to better understand *T. parva* dissemination and potential accumulation in cattle organs, a collection of tissue samples from five Friesian calves (three months old) infected with *T. parva* was tested for the presence of *T. parva* DNA. C-1419, 1420 and 1435 were infected with 5 × 10^5^ cryopreserved Ed80 *T. parva* Muguga strain (Brocklesby et al., 1961) parasites inoculated subcutaneously above the left parotid lymph node. All three animals exhibited symptoms of severe ECF including a fever (rectal temperature above 39.5 **°**C) sustained for multiple days, marked swelling of lymph nodes, weight loss and coughing. All three cattle were treated symptomatically with short acting oxytetracycline (Agrimycin 100) to control parasite proliferation but had to be euthanized 11 days post infection due to severe ECF. C-82194 was inoculated with 2 × 10^5^ cryopreserved *T. parva* P2015/1 Muguga (Brocklesby et al., 1961) parasites subcutaneously above the left pre-scapular lymph node. Infection was characterized by a sustained fever, marked lymph node enlargement, severe lymphopenia and the animal was euthanized on day 14 post infection. C-1444 was infected by subcutaneous needle inoculation of 3 × 10^4^ cryopreserved Ed64 Marikebuni (Irvin et al., 1983) strain parasites above the left pre-scapular lymph node and treated concurrently with 20 mg/kg long acting oxytetracycline (Biomycin 200). Due to the lower parasite dose and treatment with oxytetracycline, C-1444 showed no disease symptoms and no treatment was required. The animal was euthanized on day 19 post infection. Post-mortem tissue samples were taken from each calf and stored individually in sealed bags at −20 °C until used. DNA was extracted from each sample using the DNEasy blood and Tissue Kit (Qiagen) according to manufacturer’s instructions. For each tissue sample, excess blood was washed from the exterior of the tissue section with sterile distilled water. Each organ was then cut in half and three individual samples, each weighing 30 mg (10 mg for spleen samples) taken from inside the organ using a sterile razor blade. In addition, DNA was extracted from 200 μl of whole blood collected from the jugular vein from each animal on the day it was euthanized. *Theileria parva* DNA was detected in bovine blood and tissue samples by nested PCR targeting the single copy *T. parva* p104 gene (Odongo et al., 2010) (Fig. 1A). PCR using primers targeting the bovine cytochrome *b* gene was used to confirm that DNA was successfully extracted from each sample (Kirstein and Gray, 1996) (Fig. 1B). In tissue samples where *T. parva* DNA was detected by nested PCR, *T. parva* DNA concentrations were estimated by quantitative real-time PCR (Odongo et al., 2009). Concentrations were calculated as p104 copies per mg of tissue/blood. Nested PCR detected parasites in the lung, kidney, spleen, liver, lymph node, bone marrow, heart, skin and blood of C-1444 (Fig. 1A). Parasites could only be detected in one heart and two skin samples (of the three tested) and no parasite DNA could be detected in any of the three brain samples from C-1444 (Fig. 1A) suggesting that if present, they are below the threshold of detection of the test. *Theileria parva* DNA concentrations in whole blood reached quantifiable levels in the days prior to euthanasia with a maximum of 6.31 p104 copies/mg detected on day 17 post infection, two days before the animal was euthanized. The quantitative real time p104 PCR assay is less sensitive than the nested p104 PCR assay (Odongo et al., 2009) and although parasite DNA was detectable in C-1444 organs by nested PCR, they were below the limit of detection of the quantitative real-time PCR assay. *Theileria parva* DNA was detectable at quantifiable levels in all tissue samples from C-1419, C-1420, C-1435 and C-82194 including all three brain samples for each animal. Quantifiable levels of *T. parva* DNA was detected in the heart, lung, lymph node, kidney, spleen and blood of C-1419, C-1420, C-1435 and C-82194 (Table 1) with significantly higher amounts of *T. parva* DNA detected in the spleen of all three animals compared to other tissues (One-way ANOVA, p < 0.001). In addition, the bone marrow, skin and tongue samples from C-82194 all showed quantifiable levels of *T. parva* DNA (Table 1). Of the severely infected cattle C-82194 had the highest *T. parva* DNA concentration in blood and tissue samples. The ultrastructure of spleen cells from cattle infected with *T. parva* identified parasites as macroshizont stage present in the host-cell (lymphoblast) cytoplasm (Kimeto, 1981). Parasite viability was not determined in this study but successful culture of schizont infected cells from the spleen of infected cattle has been shown (Malmquist et al., 1970, Moulton et al., 1971) and it is believed that parasites proliferate indefinitely in lymphoid tissues, including the spleen of infected cattle (Moulton et al., 1984, Dobbelaere et al., 1988, Dobbelaere and Heussler, 1999). Splenic concentration of blood and removal of *T. parva* infected cells from circulation may additionally contribute to the high levels of parasite DNA observed in spleen tissues. The results of this study suggest that significant parasite accumulation in the spleen occurs during severe disease. The levels of parasite DNA detected in the circulating peripheral blood for severely infected animals was over 100 times that of asymptomatic animal C-1444. *Theileria parva* DNA could not be detected by nested PCR in the brain of C-1444 but was detectable by and quantifiable in the brain samples from C-1419, C-1420, C-1435 and C-82194 who underwent severe ECF. Cerebral theileriosis also known as ‘turning sickness’ is a neurological symptom of severe ECF infections which sometimes occurs as a result of infected lymphocytes blocking blood capillaries of the brain (Norval et al., 1992). Neurological symptoms indicative of turning sickness were not observed in any of the cattle in this study but this may have been as a result of the animals being restrained by the head for tick feeding. Skin samples from both C-1444 and C-82194 were tested for the presence of *T. parva* DNA. Piroplasm stage parasites circulating through capillaries in the skin are available for acquisition by feeding ticks. The infection level in ticks after feeding from an infected host is related to the parasitemia of the host as determined by parasitemia in a whole blood sample (Purnell et al., 1974). Only the piroplasm stage is infectious to ticks and although we do not know the proportion each parasite stage detected in this study, nymph ticks fed on C-82194 did acquire infection confirming that piroplasms were present. Table 2 summarizes the detection of *T. parva* DNA in adult ticks fed as nymphs on C-82194. Of the 25 male ticks sampled, *T. parva* DNA was detected in 13 and of the 25 female ticks sampled, 9 were infected with *T. parva*. Injection of C-82194 adult ticks with dopamine (Kaufman, 1976) induced tick salivation and *T. parva* DNA was detectable in the saliva (Table 2).

In conclusion, this retrospective study is the first to quantify *T. parva* DNA in the organs of infected cattle. Further studies should be designed to determine the mechanism of *T. parva* infected cell infiltration into organs and if this infiltration is a result of severe disease or the cause of it. The development of DNA based quantification techniques with increased sensitivity would allow parasite levels to be quantified in the organs of cattle such as C-1444 who underwent less severe infections and had overall lower parasite levels. Additionally, if long term parasite proliferation in spleen and lymph nodes does occur, sampling these organs is an intriguing avenue to pursue in an effort to identify persistent *T. parva* infections in cattle.

## Ethics statements

All experiments using animals were approved by the University of Idaho’s institutional animal use and care committee (IACUC number 2013-66). All animals were monitored closely by a registered veterinarian for symptoms of infection and general discomfort and treated accordingly. Cattle were euthanized by approved intravenous injection with sodium pentobarbital solution (Fatal Plus, Vortech Pharmaceuticals, USA).

All authors have read and approved of the submitted version of the manuscript. None of the authors have any competing interests.

## Funding

This work was supported by USAIDAID-BFS-P-13-00002, the Bill and Melinda Gates Foundation grant OPP1078791 and USDA, ARS, CRIS project #5348-32000-034-00D.

## Figures and Tables

**Fig. 1:**
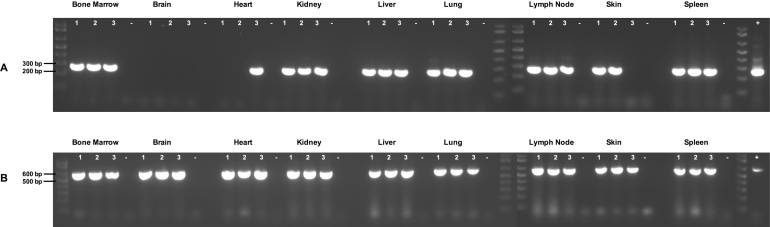
Nested PCR detection of parasites in the organs of *T. parva* infected calf C-1444. Parasites are detected in all organs except brain (A) and genomic bovine DNA was detected in each sample confirming that DNA was successfully extracted from each sample (B). Negative (−) and positive (+) controls shown.

**Table 1:** Estimation of *T. parva* DNA concentration in bovine tissue samples from calf C-1419, C-1420, 1435 and C-82194 showing a significantly higher number of p104 copies in the spleen compared to other tissue samples (One-way ANOVA, P < 0.001). Data represents average number of p104 copies per mg of tissue followed by standard deviation in parenthesis.

	C-82194	C-1419	C-1420	C-1435
Blood	1.2 × 10^4^ (2.4 × 10^2^)	6.6 × 10^2^ (2.2 × 10^1^)	9.8 × 10^2^ (8.5 × 10^1^)	5.1 × 10^3^ (6.0 × 10^1^)
Brain	5.0 × 10^2^ (7.9 × 10^1^)	6.0 × 10^1^ (3.7)	2.3 × 10^2^ (8.3 × 10^1^)	6.0 × 10^2^ (2.2 × 10^2^)
Liver	2.9 × 10^5^ (9.3 × 10^4^)	5.3 × 10^3^ (2.7 × 10^3^)	1.4 × 10^4^ (7.2 × 10^3^)	6.5 × 10^4^ (2.2 × 10^4^)
Lung	2.4 × 10^5^ (1. ×10^5^)	1.1 × 10^4^ (4.4 × 10^3^)	2.5 × 10^4^ (4.7 × 10^3^)	1.6 × 10^4^ (1.0 × 10^4^)
Kidney	2.9 × 10^5^ (1.6 × 10^5^)	1.3 × 10^4^ (6.7 × 10^2^)	5.4 × 10^3^ (5.7 × 10^3^)	5.1 × 10^4^ (7.3 × 10^3^)
Spleen	8.7 × 10^5^ (3.6 × 10^5^)	1.4 × 10^5^ (1.5 × 10^4^)	3.0 × 10^5^ (1.0 × 10^5^)	2.6 × 10^5^ (9.2 × 10^4^)
Lymph node (LPSG)	2.8 × 10^5^ (2.3 × 10^5^)	Not sampled	1.4 × 10^4^ (5.9 × 10^3^)	1.2 x ×10^3^ (2.5 × 10^2^)
Lymph node (RPSG)	4.1 × 10^4^ (3.7 × 10^4^)	Not sampled		
Bone Marrow	1.6 × 10^4^ (2.6 × 10^3^)			
Heart	8.4 × 10^3^ (2.2 × 10^3^)			
Skin	2.1 × 10^3^ (1.6 × 10^2^)			
Tongue	3.2 × 10^3^ (1.1 × 10^3^)			

**Table 2:** Detection of *T. parva* DNA in adult ticks fed as nymphs on acutely infected calf C-82194.

	Number infected ticks	Average number of sporozoites per tick^a^	Average number of sporozoites detected in saliva^a^
Male	13 of 21	3.2 × 10^5^ (1.3 × 10^5^)	2.0 × 10^4^ (6.0 × 10^3^)
Female	6 of 23	3.2 × 10^5^ (1.1 × 10^5^)	2.4 × 10^4^ (1.1 × 10^4^)

a Number of sporozoites determined by quantitative real-time PCR and represented as population mean (standard error).
